# Morphology and electronic structure of nanoscale powders of calcium hydroxyapatite

**DOI:** 10.1186/s11671-015-0770-1

**Published:** 2015-02-05

**Authors:** Nataly Kurgan, Volodymyr Karbivskyy, Vasyl Kasyanenko

**Affiliations:** G.V. Kurdymov Institute for Metal Physics of the NAS of Ukraine, blvd. Vernadsky, 36, Kiev, 03680 Ukraine; Vinnytsia National Technical University, 95 Khmelnytske shose, Vinnytsia, 21021 Ukraine

**Keywords:** Atomic force microscopy, Hydroxyapatite, Morphology, Electronic structure, 87.64.Dz, 87.64.kj, 87.80.Lg

## Abstract

Atomic force microscopy, infrared spectroscopy and NMR studied morphological and physicochemical properties of calcium hydroxyapatite powders produced by changing the temperature parameters of synthesis. Features of morphology formation of calcium hydroxyapatite nanoparticles with an annealing temperature within 200°C to 1,100°C were determined. It is shown that the particle size of the apatite obtained that annealed 700°C is 40 nm corresponding to the particle size of apatite in native bone. The effect of dimension factor on structural parameters of calcium hydroxyapatite is manifested in a more local symmetry of the PO_4_^3−^ tetrahedra at nanodispersed calcium hydroxyapatite.

## Background

Bioactive materials based on calcium hydroxyapatite (Ca_10_(PO_4_)_3_(OH)_2_, HAP), the chemical composition and structure of which approaches the mineral component of bones, are widely used in medical practice over the past 20 years. All the time, the researches continue to work for improvement of these materials [1-4]. However, the prospect of their application may not be quite complete without clarifying the relationship of morphological, physical and chemical properties, and atomic architecture surfaces of these materials because biocompatibility is dependent not only on their chemical composition but also to a large extent on the morphology of the particles, which affect the interaction of mineral constituent of bones with the organic matrix. In view of the fact that such material should be included in the metabolism of the body and be replaced with a full bone tissue, it remains relevant to obtain nanodispersed calcium hydroxyapatite with parameters that match the parameters of particle maximum mineral component of bones [5-8]. This article presents the results of a study of morphological and physicochemical properties of calcium hydroxyapatite powders obtained at various temperature parameters of synthesis.

## Methods

### Preparation of calcium hydroxyapatite

For preparation of hydroxyapatite powder, calcium nitrate tetrahydrate and diammonium hydrogen phosphate (reagent grade) were used as calcium and phosphorus precursors, respectively. Both reagents were purchased from Merck, Darmstadt, Germany. Urea (R&M Chemicals, Edmonton, UK) was used as gelling and ammonium donor agent. EDTA (Merck) was used as a chelating agent to prevent an immediate precipitate formation calcium ion in the course of gel formation. The reaction was conducted in a basic solution using ammonium solution (R&M Chemicals) as a solvent. Ammonium solution was heated at 60°C, and EDTA (182 g) was added while stirring until it dissolved. Into this, 200 mL of aqueous solution of 128 g calcium nitrate tetrahydrate was poured. Diammonium hydrogen phosphate (39.83 g) and urea (45.20 g) were subsequently added. The mixture was heated at 100°C for 3 to 4 h. The obtained gel was dried at 150°C under ambient static air and subsequently subjected to an 200°С, 500°С, 700°С, 900°С, 1,000°С, and 1,100°С calcination under flowing air for 1 h. The powder was examined by X-ray diffraction techniques to determine the phases formed. It was observed that Ca/P molar ratio is 1.667.

### Morphology investigation

Morphology of hydroxyapatite powder has been investigated by scanning probe microscope JSPM-4500/4610 (JEOL, Tokyo, Japan) using atomic force microscopy. Imaging parameters were as follows: image size 90.0 × 90.0 nm, image height 12.2 nm, and exposure time at 83.33 μs. As the scanning probe made of cantilever with a diamond tip of the NSG-10-DLC. Operational vacuum was no worse than 10^−7^ Pa.

### Infrared spectroscopy investigation

IR spectra were obtained on double-beam spectrophotometer SPECORD M80 (Carl Zeiss, Jena, Germany). To provide the study, the samples were prepared by compressing into the mixed tablets of the compound and powdered KBr. Tablets of pure KBr powder were also prepared to measure of the sample phonon transmission. Transmission spectra were recorded in the absorption range of the tetrahedral sublattice PO_4_^3−^ with vibrational modes lying in the 4,000 to 400 cm^−1^ range.

In the region of slow electromagnetic waves, sharp KBr powder absorption begins; therefore, the measurement in the range <400 cm^−1^ is not correct. The measurements were made at a constant level of signal/noise ratio in the whole measurement range. To optimize the recording of the spectra, an electromagnetic radiation attenuator was also installed in comparison channel, so that the absorption background level of KBr pellet approaches to 80%. During the measurements, the sample chamber was additionally blown through with dry air for thorough drying of water vapor.

### NMR investigation

NMR spectra were recorded on a spectrometer Avance 400 (Bruker, Ettlingen, Germany) at room temperature. NMR spectra were obtained from rotation of the sample under the magic angle (MAS NMR) at frequencies of 10 and 15 kHz, which eliminates the effects of anisotropy and dipole-dipole interaction, but does not exclude the quadrupole interaction of the second order.

## Results and discussion

### Morphology of nanoscale powders

**Figure 1:**
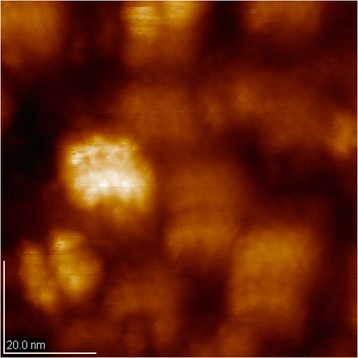
**AFM image of HAP samples obtained at the temperature of 200°C.** Transition of amorphous condensate-crystal phase is observed in samples obtained at 200°C. There are clusters of crystal phase size 10 to 15 nm in an amorphous matrix. Image size is 90.0 × 90.0 nm.

It should be noted that the powders obtained by sol-gel method formation of apatite are similar with the formation of apatite in bone native mechanism: colloidal solution-amorphous condensate-crystal phase. Kinetic chain synthesis involves the transformation of sol in the gel, condensation precipitate, rinsed with water, and drying. Since the composition of the amorphous shell in particles cannot be determined, we can conclude only a disordered agglomeration of the molecules. Transition of amorphous condensate-crystal phase is observed in samples obtained at 200°C. Simultaneous formation of amorphous particles begins with the process of crystallization, i.e., begins with the formation of the long-range order within the amorphous phase. There are clusters of crystal phase size 10 to 15 nm in an amorphous matrix (Figure 1).

**Figure 2:**
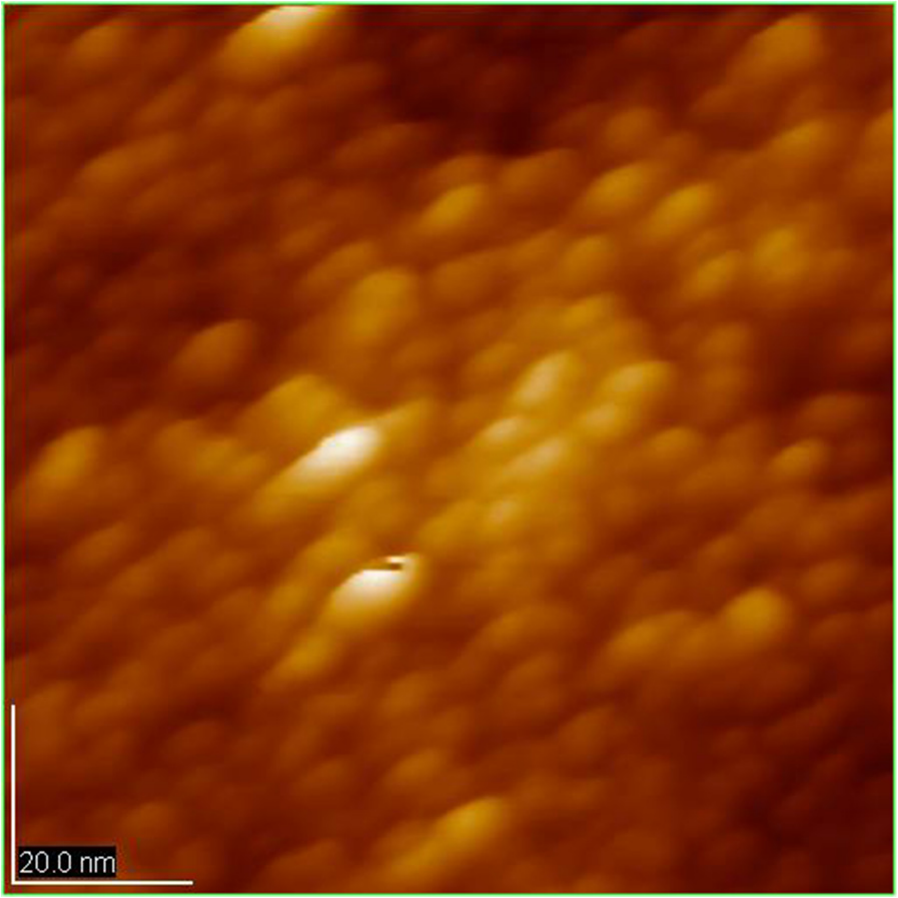
**AFM image of HAP samples obtained at the temperature of 500°C.** There has been an increase in the size of the crystal phase clusters. The particle size is 15 to 20 nm. Image size is 90.0 × 90.0 nm.

When the temperature reaches up to 500°C, it shows an increase in the size of the crystal phase clusters. Aspiration systems reduce the surface energy leads to the activation of aggregation processes: coagulation and coalescence of clusters, resulting in an increase in grain size crystal phase. In Figure 2 shows a picture of nanoparticles obtained at 500°C temperature conditions in which the collective process of recrystallization completed.

**Figure 3:**
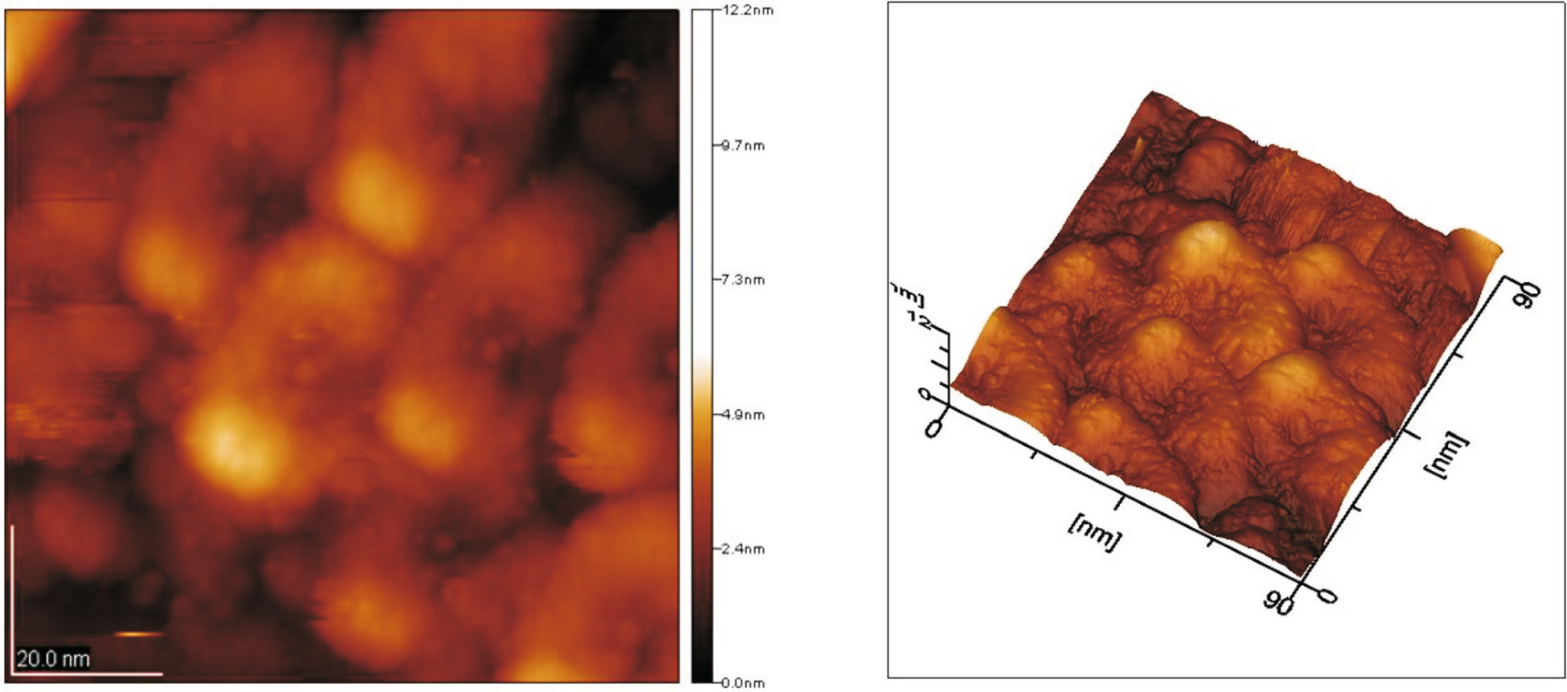
**AFM image of HAP samples obtained at the temperature of 700°C.** The particle size of the apatite is 40 nm. There is a horseshoe shape of the particles. Image size is 90.0 × 90.0 nm; image height is 12.2 nm.

This process continues up to a temperature of 700°C. It should be noted that the most rapid growth of crystals occurs in the temperature range of 600°C to 700°C [1-3]. The particle size of the apatite obtained by annealing at 700°C is 40 nm (Figure 3), corresponding to a particle size of apatite in native bone. There is a horseshoe shape of the particles. However, the bone apatite particles are needle shaped. The horseshoe shape is due to the application in the synthesis of drying powder in which the ends of the particles are not committed and bent under the influence of surface tension, creating a shape that resembles a horseshoe.

Annealing at temperatures above 900°C leads to complex processes of mass transfer and formation of crystals of different morphology and particle size range from 200 to 500 nm.

The crystallization process takes place before the full absorption of the crystalline phase of amorphous matrix. Structural changes in the crystal lattice during the transition amorphous condensate-crystal phase can mostly be traced back to change the width of the vibrational bands in the spectra of crystalline lattice.

### IR and NMR spectroscopy of nanodispersed apatites

It is known [1] that spectrum of initial phosphate hydroxyapatite is characterized by two intense groups of bands around 1,040 and 570 cm^−1^. Nine possible variations of XO_4_ group in the case of equivalence of all X-O bonds, i.e., tetrahedral symmetry *T*_d_, give only two bands of IR spectrum: one band of *v*_3_ vibrations and another of *v*_4_ ones, the vibrations *v*_2_ and *v*_1_ being active only in Raman spectra [9].

If only three of four bonds are equal (symmetry *C*_3v_), then total symmetric vibration *v*_1_ becomes active, and the splitting of three degenerate vibrations *v*_3_ and *v*_4_ is removed partially. When there is a non-equivalence of the two X-O bonds with respect to the other two (point symmetry *C*_2v_) degenerations of vibrations, *v*_3_ and *v*_4_ are removed completely. Finally, in the case when all four bonds are different (symmetry *C*_s_), there is another change in the spectrum - the removal of doubly degenerated vibration *v*_2_ [9,10]. Thus, widening the bands that correspond to vibrations of the PO_4_ groups indicates a decreasing of surrounding symmetry of the (РО_4_)^3−^ anion in apatite sublattice.

Study on IR spectra of samples (Table 1) revealed a lower bandwidth, characterizing PO_4_^3−^ tetrahedra for nanodispersed samples one to three, indicating a higher local symmetry of the PO_4_^3−^ tetrahedra in nanodispersed apatite in comparison with macrosamples four to five.

**Table 1:** **Half width of the absorption band** ***ν***_**3**_ **of studied compounds**

**Temperature of the HAP samples annealing,** ***T*** **,°C**	**Half width of the absorption band at 1,040 cm** ^**−1**^ **, characterizing PO**_**4**_^**3−**^ **tetrahedra**
200	130
500	116
700	120
900	193
1,000	>272

**Figure 4:**
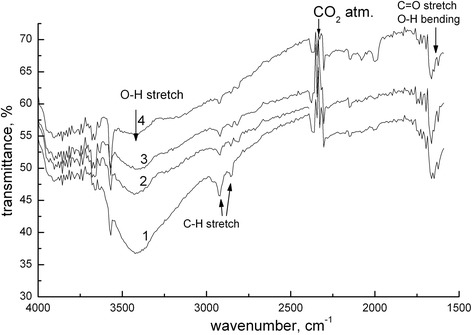
**The presence of organic impurities in the samples according to IR spectroscopy.** Annealing temperature samples: 1 - 200°C; 2 - 500°C; 3 - 900°C; 4 - 1,000°C. Transmission spectra were recorded in the absorption range in the 4,000 to 400 cm^−1^.

It should be noted that the presence of impurity atoms and other defects significantly affects vibrational infrared spectra. In the investigated samples are the presence of CO_2_ from the atmosphere and a small amount of residual organic reaction (Figure 4). Thermal annealing of HAP leads to changes in the hydroxyl sublattice: the number of OH^−^ ion decreases with increasing annealing temperature, as evidenced by the decrease in the intensity of the bands at approximately 3,400 and 2,900 cm^−1^, which correspond to vibration stretching of O-H and C-H bonds (Figure 4).

**Figure 5:**
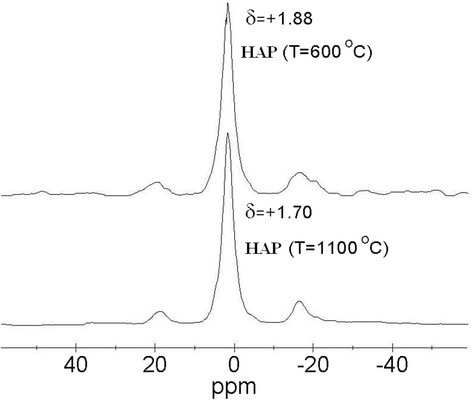
^**31**^ **P-NMR spectra of compounds.**

^31^Р-NMR spectra at annealing temperature increase are offset of the dominant signal in the direction of a strong field of *δ* = 1.88 to *δ* = 1.70 ppm (Figure 5). A significant offset of the ^31^Р spectrum to lower frequency range (Figure 5) with annealing temperature increases points to an increase in magnetic shielding of the phosphorus nuclei, which in this case means an increase in electron density on the phosphorus atoms.

To determine the effects of annealing modes on HAP samples has been used in NMR method (Figure 5). It is known that ^31^P-NMR line quite narrows for the reference compound (phosphoric acid), whereas in the HAP, its intensity decreases, increases its half width, and maximum shifted to higher frequencies (approximately 850 Hz).

## Conclusions

This is a study of crystal growth mechanism of calcium hydroxyapatite in the synthesis of sol-gel method with variation of temperature parameters of synthesis within 200°C to 1,100°C. The origin of crystal phase occurs in the amorphous matrix by clustering, growth, and subsequent coagulation grains with the formation of crystals in a wide range of dispersion.

Temperature determines the kinetics of structural transformation of amorphous phase in the crystal phase, which in turn affects the characteristics of the particle substructure. It is shown that the particle size of the apatite obtained that annealed 700°C is 40 nm corresponding to the particle size of apatite in native bone.

The effect of dimension factor on structural parameters of calcium hydroxyapatite is manifested in a more local symmetry of the PO_4_^3−^ tetrahedra at nanosized calcium hydroxyapatite. Thermal annealing of HAP leads to changes in the hydroxyl sublattice: of number OH^−^ ions decreases with increasing annealing temperature. A significant offset of the ^31^P spectrum to a low frequency range with increasing annealing temperature indicates an increase of electron density on the phosphorus atoms.
